# Targeting Low-arsenic Groundwater with Mobile-phone Technology in Araihazar, Bangladesh

**Published:** 2006-09

**Authors:** A. van Geen, M. Trevisani, J. Immel, Md. Jakariya, N. Osman, Z. Cheng, A. Gelman, K.M. Ahmed

**Affiliations:** ^1^ Lamont-Doherty Earth Observatory of Columbia University, Palisades, NY, 10964, USA; ^2^ Department of Economic and Statistical Sciences, University of Trieste, Trieste, Italy; ^3^ John Immel Consulting, East Moriches, NY 11940; ^4^ Research and Evaluation Division, BRAC, 75 Mohakhali, Dhaka 1212; ^5^ Bangladesh Arsenic Mitigation and Water Supply Program, Dhaka 1000; ^6^ Department of Statistics, Columbia University, New York, NY, USA; ^7^ Department of Geology, University of Dhaka, Dhaka 1000

**Keywords:** Arsenic, Water supply, Water pollution, Dugwells, Cell-phone technology, Bangladesh

## Abstract

The Bangladesh Arsenic Mitigation and Water Supply Program (BAMWSP) has compiled field-kit measurements of the arsenic content of groundwater for nearly five million wells. By comparing the spatial distribution of arsenic inferred from these field-kit measurements with geo-referenced laboratory data in a portion of Araihazar upazila, it is shown here that the BAMWSP data could be used for targeting safe aquifers for the installation of community wells in many villages of Bangladesh. Recent experiences with mobile-phone technology to access and update the BAMWSP data in the field are also described. It is shown that the technology, without guaranteeing success, could optimize interventions by guiding the choice of the drilling method that is likely to reach a safe aquifer and identifying those villages where exploratory drilling is needed.

## INTRODUCTION

**Fig. 1.**
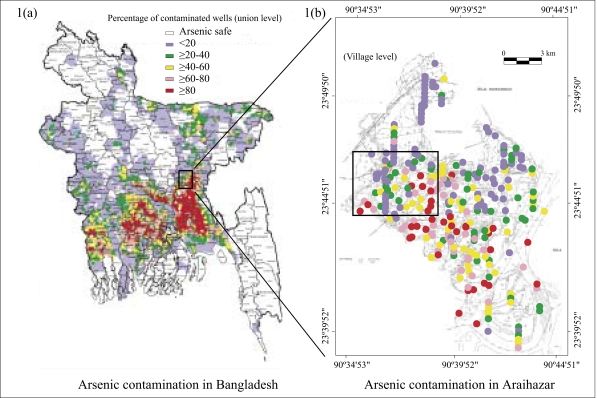
(a) Map of available BAMWSP results, colour-coded according to proportion of unsafe wells at upazila level (http://www.bamwsp.org); (b) expanded view of Araihazar upazila showing the proportion of unsafe wells in 297 villages using the same colour scheme. The coordinates of a central location of 109 villages were determined with a hand-held Global Position System receiver. For the remaining villages, a central location of the Mouza to which they belong was read from the upazila map. Whenever a Mouza has more than one village whose precise location was not determined, symbols representing each village were lined up vertically below a central Mouza location. The rectangle delineates the Columbia University and University of Dhaka's study area enlarged in Fig. 2

Elevated concentrations of arsenic in groundwater of Bangladesh were first reported in 1993 (1). Broader-scale sampling and laboratory testing subsequently established the regional extent of the problem (1, 2). These surveys, drawn from a small fraction (∼0.1%) of existing wells, guided the selection of a subset of 269 of 490 upazilas where a blanket survey of million tubewells, coordinated under the Bangladesh Arsenic Mitigation and Water Supply Program (BAMWSP), was carried out by various organizations, including the United Nations Children's Fund (UNICEF). For logistical reasons, these blanket surveys have relied on field-kits rather than laboratory measurements. Nearly five million field-kit results, compiled by the BAMWSP, as of May 2005, provide the most extensive and detailed representation of the spatial distribution of arsenic in groundwater of Bangladesh to date (Fig. 1a). Maps drawn based on the BAMWSP data to show the proportion of unsafe wells in each surveyed upazila are broadly consistent with previous surveys (we refer in this paper to the status of a well relative to the Bangladesh standard of 50 μg/L arsenic in drinking-water, unless the World Health Organization (WHO)'s guideline value of 10 μg/L is specified). The purpose of the present study was to show that the large quantity of field-kit data compiled by the National Arsenic Mitigation Information Center (NAMIC; http://www.bamwsp.org/search.htm) and disseminated by the BAMWSP could be used for targeting those aquifers that are low in arsenic when examined at the village level. The paper also describes how the BAMWSP data can be accessed and updated from the field using mobile-phone technology that is readily available in Bangladesh.

**Fig. 2.**
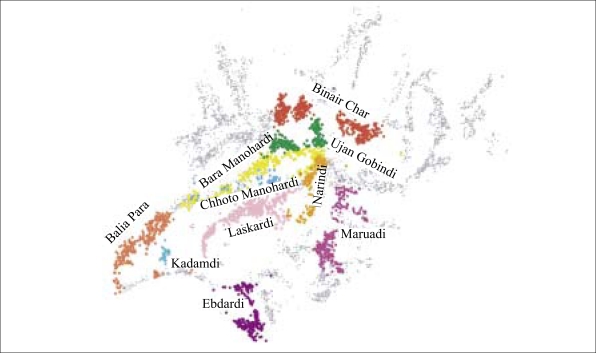
Expanded view of 10 villages in Araihazar surveyed by Columbia University and BAMWSP identifying by colour the location of wells belonging to different villages and

BAMWSP=Bangladesh Arsenic Mitigation and Water Supply Program

Despite its scale, the impact of the blanket-testing campaign is not well-known because responses of households to well-testing have rarely been quantified. Response surveys, conducted in Columbia University's study area of Araihazar upazila, indicate that blanket-testing led roughly half the affected households to switch their consumption of water away from those wells that were identified as unsafe (3–5). While this represents a very significant benefit of well-testing, the same observations also showed that a significant number of households did not stop drinking water from their wells after learning that it was unsafe. The premise of this study is that the installation of one (or several) safe community well(s) in the most-affected villages holds particular promise to reach those remaining households. Our experiences in Araihazar suggest that the benefit of such installations can be direct in the sense that safe water is provided to households living within walking distance of a community well, but also indirect because a successful installation serves as a guide to the installation of safe private wells to the same depth that are likely to follow (5, 6).

Safe community wells could over time be connected to the rural piped-water supply systems, although we believe that priority should be given to providing point-sources of safe water throughout the country. We do not claim that alternative approaches to arsenic mitigation, such as shallow dugwells, arsenic removal from groundwater, treatment of surface water, or collection of rainwater, have no role to play in Bangladesh (7, 8). We merely point out that these alternatives are a more radical departure from the current household practice of relying primarily on shallow tubewells to obtain drinking-water compared to switching over to a deeper community well.

An argument against continued reliance on tubewells of any sort is that concentrations of arsenic in groundwater could increase over time. Various mechanisms that could lead to such an increase have been proposed but have not been substantiated by systematic observations (9–11). The most troubling evidence of increases in arsenic concentrations over time comes from West Bengal where a same set of wells were sampled five years apart (10, 12). By and large, however, published results indicate that if concentrations of arsenic change over time, they probably affect only a minority of wells in Bangladesh (1, 13–15). Volumetric considerations also suggest that increases in arsenic concentration in deeper aquifers in response to increased withdrawals with hand-pumps is unlikely (16). Contamination in deeper aquifers with shallow groundwater elevated in arsenic could occur if mechanized pumping of much larger volumes of water for irrigation were to tap deeper aquifers. More likely than large-scale contamination of deep aquifers appears to be local contamination due to leakage of shallow groundwater along an improperly installed well (14, 17). Such faulty construction may partly explain scattered but significant trends showing an increase in arsenic concentration with age of well (1, 18–20). It is principally for this reason that all wells from which water is regularly drawn for drinking or cooking should be tested periodically for arsenic.

Since the rural population of Bangladesh is likely to rely on groundwater for the foreseeable future, how could the BAMWSP data be used more effectively for installing safe wells? This is demonstrated in this paper by first reviewing the sample collection and analysis procedures followed in Araihazar by the Columbia University and University of Dhaka (CU/UD) team and BAMWSP. Technical aspects of down- and uploading well data using mobile phones are also reviewed. The results section starts by comparing the spatial distribution of arsenic in groundwater inferred from the BAMWSP field-kit measurements and geo-referenced laboratory data in 10 villages of Araihazar. Predictions based on a simple algorithm developed by Gelman *et al.* (21) to identify the likely transition to a safe aquifer are shown to compare favourably with the actual depths of 23 community wells installed in the area. The discussion expands the analysis by considering the BAMWSP data for the ∼300 villages in the entire upazila. Examples drawn from the BAMWSP data are then used for showing how existing mobile-phone technology can be used for guiding the installation of safe wells.

## MATERIALS AND METHODS

### Well-water collection and analysis

In total, 6,500 tubewell-water samples were collected from 60 villages distributed over a 25-sq km area in 2000–2001 by CU/UD. The location of each sampled well was determined with hand-held Global Positioning System (GPS) receivers; well depth was estimated based on the number of PVC pipes that went into the construction of the well, as recalled by the owner. Van Geen *et al.* provided the details of the quality-control procedures followed for analysis by graphite-furnace atomic absorption of dissolved arsenic (18). Placards reporting the results were posted on the wells a first time in 2001 and a second time in 2004 (5).

Approximately, 30,000 tubewells were tested throughout Araihazar with the Hach-kit by teams of workers from non-governmental organizations (NGOs) hired by the BAMWSP in 2002–2003. The spout of each well was painted red or green for test results corresponding to an arsenic concentration of ≤50 and >50 μg/L respectively. In addition to test results reported as one of several ranges of arsenic concentrations, the depth of each well and the names of the village and higher administrative units were recorded on paper forms and manually entered in a database in Dhaka.

Rahman *et al.* have challenged the quality of arsenic measurements obtained with field-kits (22), but this is no longer a major issue. Of the nearly five million tests compiled by the BAMWSP, roughly half were conducted using the Hach field-kit that became available in 2001. Inter-calibration with laboratory measurements showed that field workers hired by the BAMWSP correctly identified the status of the vast majority of wells compared to the Bangladesh standard of 50 μg/L for arsenic in drinking-water with the Hach-kit in Araihazar (13). The reliability of previous measurements conducted with other kits may have been lower (22), but as we show in this paper, the impact of occasionally poor measurements is limited when trying to identify spatial patterns for the installation of community wells.

### Installation of community wells

During January 2002–October 2003, 23 community wells were installed in eight of the 10 villages for which complete coverage with both laboratory and field-kit data is available (An additional 27 community wells were installed in surrounding villages). All but three of these wells are less than 230-ft (69-m) deep, and a local drilling team installed these wells using the entirely manual ‘hand-flapper’ method. An outside team of drillers installed the three community wells ranging in depth from 340 to 490 feet (102–147 m) using a hydraulic ‘dunkin’ pump and a tall bamboo-rig. In most cases, drilling continued until orange-brown aquifer material typically associated with low concentrations of arsenic was reached (16, 23, 25). Immediately after installation, the community wells were tested for arsenic with the Hach-kit and later in the laboratory by high-resolution inductively-coupled plasma mass spectrometry (HR ICP-MS), a method that is considerably more accurate than graphite-furnace atomic absorption (GFAA) at low concentrations of arsenic (24).

### Algorithm for estimating safe-depth thresholds

Gelman *et al.* developed a search algorithm for estimating safe-depth thresholds for spatial clusters of ∼75 wells (21). In this paper, we replace the previous terminology with the expression ‘start-depth’ to emphasize the possibility of having to drill deeper before a safe aquifer is reached. The algorithm starts from the deepest wells and identifies a start-depth below which one can be reasonably confident of the presence of groundwater that is low in arsenic. A single aberration or outlier, which could reflect an incorrect depth or arsenic entry, is accommodated by the algorithm. To estimate the probability that a well-drilled below the start-depth is safe, the algorithm follows an approximate Bayesian approach that takes into account the number of safe wells below the start-depth and occasional unsafe wells (21). For those clusters of wells where no start-depth can be identified, the algorithm produces a minimum depth below which the start-depth still needs to be determined. In the present study, the same search algorithm was used for analyzing clusters of wells defined by village name rather than geographic position.

### Mobile phone and server technology

The first technology that was tested in Bangladesh relied on client software that was purposely developed and required a Java-enabled mobile phone. Such phones are available in Bangladesh but are not widely used because these, and access to the Internet that these phones provide, are relatively expensive. The technology was modified in November 2005 to allow users to download and update the BAMWSP data through the Short Message Service (SMS) offered by local providers. SMS is popular throughout Bangladesh, works on less-expensive phones, and is more reliable than the Internet protocol and is less also expensive. In the present configuration, a mobile phone is connected to a laptop acting as a server. SMS requests are handled by jSMSEngine and mySQL, a robust open software model database server. The software queries the database, runs a search algorithm programmed in R that estimates the start-depth, and sends the response back to the mobile phone via SMS. An Internet-based version is available at http://www.ldeo.columbia.edu/welltracker/. In remote areas, mobile-phone service is sometimes unavailable. In that situation, users can save the message and re-send when the network becomes available.

## RESULTS

### Comparison of laboratory and field-kit data

**Fig. 3.**
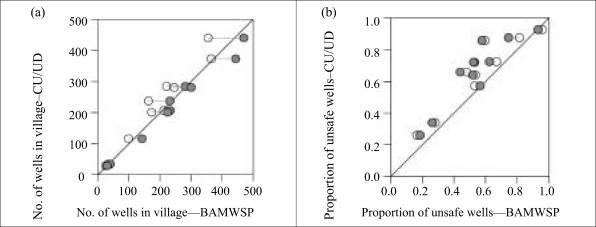
Comparison of (a) number of safe wells for 10 overlapping villages under Columbia University and University of Dhaka and Bangladesh Arsenic Mitigation and Water Supply Program and (b) proportion of safe wells for the same 10 villages. Open symbols show results for wells reported to be installed through 2000, and closed symbols show results for the entire BAMWSP dataset

The depth distribution of arsenic inferred from the two datasets is compared for 10 villages containing 30–400 wells each that were covered in their entirety by both the surveys (Fig. 2). The CU/UD and BAMWSP surveys inventoried 2,205 wells in 2000–2001 and 2,396 wells in 2002–2003 in these villages respectively. The increase in the number of wells is consistent with previous observations (5). The generally higher proportion of unsafe wells reported by the CU/UD surveys—higher by 12±9% on average (Fig. 3)—also matches the observation that most inconsistencies between the two methods reflect an under-reporting of unsafe wells by the BAMWSP survey for wells containing arsenic of 50–100 μg/L (13).

BAMWSP=Bangladesh Arsenic Mitigation and Water Supply Program;
CU=Columbia University;
DU=University of Dhaka

**Fig. 4.**
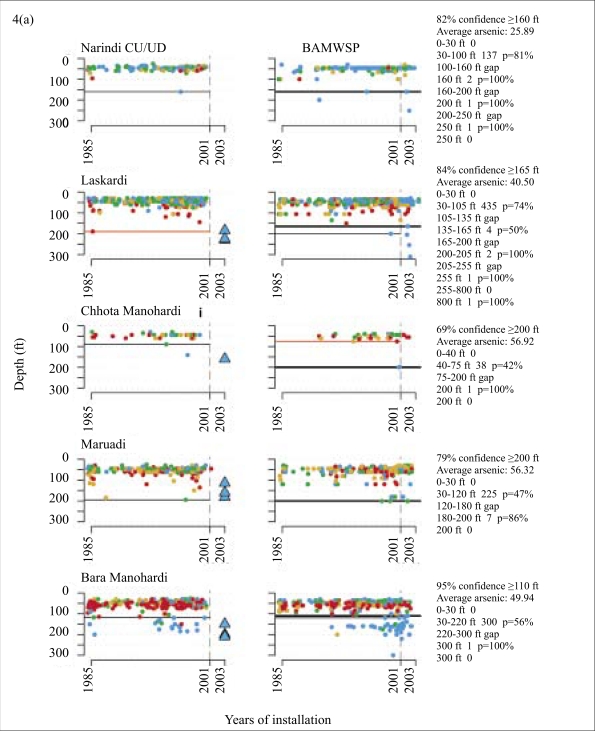

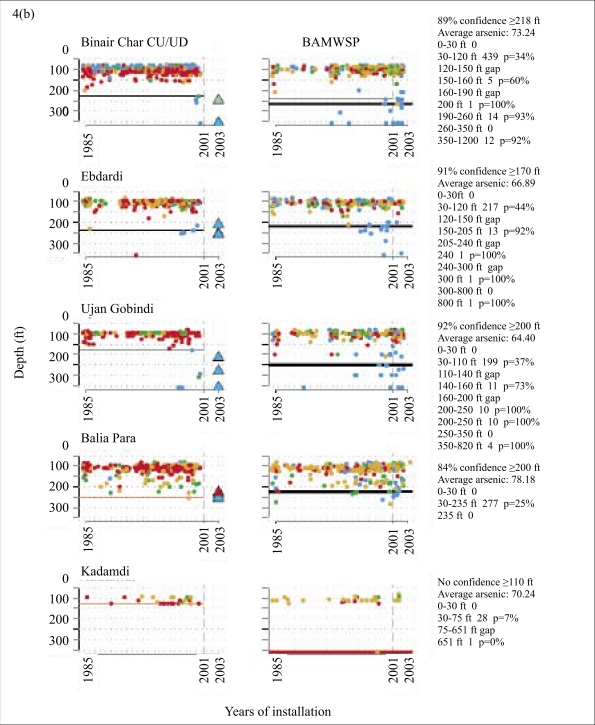
Comparison of CU/UD and BAMWSP results for 10 individual villages as a function of depth and year of installation, together with output from the safe-depth algorithm. The actual depths selected for the installation of 23 community wells are shown by coloured triangles

The depth distribution of arsenic inferred from the two surveys was broadly consistent, taking into account that new wells were installed after the CU/UD survey (Fig. 4). In the relatively small village of Kadamdi, for instance, both the surveys indicated a predominance of unsafe wells and shallow wells (<80 ft [24 m]). Both the surveys also indicated a predominance of shallow wells in the small villages of Narindi and Chhota Manohardi, although with a somewhat higher proportion of safe wells. These distributions contrasted sharply with the larger village of Bara Manohardi where, for instance, both the surveys showed unsafe wells extending to ∼100 ft (30 m) and 20–30 wells beyond this depth that are mostly safe. The situation is analogous in the relatively large villages of Edbardi and Ujan Gobindi. Significantly, the BAMWSP survey indicated that a number of deeper, safe wells were installed after the first survey was completed in these three villages and in Binair Char and Laskardi (Fig. 4).

BAMWSP=Bangladesh Arsenic Mitigation and Water Supply Program;
CU=Columbia University;
UD=University of Dhaka

### Start-depths and community wells

It is instructive to compare the depth of installed community wells with the start-depths even if the drilling was conducted before the algorithm was available. In Laskardi village, for instance, the 2000–2001 survey could identify only a minimum depth of 190 ft (57 m) for the start-depth (Fig. 4a). Three community wells meeting the WHO guideline value of 10 μg/L for arsenic were subsequently installed at depths ranging from 185 to 230 ft (56–69 m; Table 1). These are probably some recent and deeper wells that were subsequently tested by the BAMWSP in the same village, even if the reported depths did not exactly match (Fig. 4a). On the basis of these new wells, a revised start-depth of 165 ft (50 m) with a probability of 0.84 is estimated by the algorithm (Table 2). This is a situation where the installation of a few wells changed the perception of the options available to households by indicating that a safe aquifer is readily accessible using the local ‘hand-flapper’ drilling method. The situation evolved in a similar fashion between the two surveys for the villages of Bara Manohardi (Fig. 4a) and Edbardi (Fig. 4b), although, in those cases, the algorithm could already identify a start-depth ranging from 120 to 200 ft (36–60 m) based on the data collected during 2000–2001. In several cases, the exclusion of an occasional outlier by the algorithm was justified by subsequent installations.

**Table 1.** Characteristics of 23 community wells installed in 8 villages of Araihazar, including arsenic, manganese, iron, and sodium content of well-water (*re-installed well)

Village	CW ID	Installation	Latitude	Longitude	Depth	As	Mn	Fe	Na
					(ft)	(μg/L)	(g/L)	(mg/L)	(mg/L)
Balia Para	38	Oct 03	23.7692	90.5851	180	349	0.6	2.2	467
“	38*	Mar 05	23.7692	90.5851	180	15	0.6	1.9	465
“	8	Apr 03	23.7737	90.5946	182	1	0.6	0.1	373
“	18	Jul 03	23.7740	90.5923	205	3	0.0	0.1	193
“	17	Jul 03	23.7715	90.5908	208	1	0.2	0.6	632
Bara Manohardi	9	May 03	23.7861	90.6214	150	1	0.2	0.0	81
“	21	Sep 03	23.7793	90.6080	200	1	0.3	0.2	174
“	22	Sep 03	23.7840	90.6133	205	1	0.1	0.5	30
Binair Char	10	May 03	23.7899	90.6361	200	18	0.0	3.0	181
“	34	Oct 03	23.7902	90.6311	340	3	0.1	1.5	23
“	46	Oct 03	23.7918	90.6182	420	3	0.0	1.0	22
Chhota Manohardi	24	Sep 03	23.7814	90.6178	160	2	0.1	0.3	47
Edbardi	37	Oct 03	23.7628	90.6114	160	2	0.3	10.5	335
“	13	Jul 03	23.7550	90.6125	206	1	0.1	0.2	198
“	15	Jul 03	23.7562	90.6101	208	1	0.2	0.1	332
Laskardi	F	Jan 02	23.7736	90.6045	185	2	0.5	0.9	227
“	26	Sep 03	23.7751	90.6199	225	4	0.5	3.9	151
“	25	Sep 03	23.7762	90.6171	230	4	0.2	0.3	133
Maruadi	28	Sep 03	23.7695	90.6263	114	1	1.1	1.0	25
“	27	Sep 03	23.7679	90.6291	160	1	1.1	0.4	10
“	12	May 03	23.7761	90.6324	182	3	0.3	0.2	15
Ujan Gobindi	23	Sep 03	23.7874	90.6174	165	1	0.5	0.2	26
“	33	Oct 03	23.7875	90.6267	230	3	0.1	2.5	58
“	36	Sep 03	23.7907	90.6264	490	3	0.2	1.9	27

As=Arsenic;

CW=Community well;

Fe=Iron;

ID=Identification;

Mn=Manganese;

Na=Sodium

**Table 2.** Properties of 10 villages of Araihazar surveyed twice in their entirety by Columbia University and University of Dhaka and by Bangladesh Arsenic Mitigation and Water Supply Program

Village	CU/UD		BAMWSP			
	No. of wells	% unsafe wells	No. of wells	% unsafe wells	Safe-depth (ft)	Probability
Narindi	116	0.26	141	0.18	160	0.82
Laskardi	373	0.34	443	0.26	165	0.84
Chhota Manohardi	35	0.57	39	0.56	200	0.69
Maruadi	236	0.64	232	0.52	200	0.79
Bara Manohardi	282	0.66	301	0.44	110	0.95
Binair Char	408	0.70	470	0.62	218	0.89
Edbardi	206	0.72	233	0.52	170	0.91
Ujan Gobindi	202	0.86	224	0.58	200	0.92
Balia Para	285	0.88	277	0.74	175	0.84
Kadamdi	28	0.93	29	0.93	>651	

BAMWSP=Bangladesh Arsenic Mitigation and Water Supply Program;

CU=Columbia University;

UD=University of Dhaka

The situation was not as clear-cut for several other villages. In Maruadi, for instance, the installation of three community wells shallower than 200 ft (60 m) was successful. Yet, the algorithm yielded a start-depth of 200 ft (60 m) with a probability of 0.79 on the basis of the subsequent BAMWSP survey (Fig 4a). The reason is the presence of a significant number of unsafe wells in the 100–200-ft (30–60-m) range reported by both the surveys. This could be due to errors in data collection and entry or, more likely, significant variability in the sub-surface geology of this particular village. This may be the case also in the villages of Binair Char and Ujan Gobindi (Fig. 4b). In each of these villages, at least one safe community well was successfully installed at a depth that was consistent with the start-depth identified by the 2000–2001 data. However, for one other location in each of these villages, drilling had to be extended beyond 300 ft (90 m) by a drilling team using a ‘dunkin’ pump to reach the orange-brown aquifer material associated with low concentrations of arsenic in groundwater. The variability of the sub-surface geology, therefore, appears to be particularly complex in these two villages.

A different situation was encountered in Balia Para. In that village, four community wells, installed in the 180–220-ft (54–66-m) depth range initially all met the WHO guideline for arsenic at 10 μg/L (Fig. 4b). Then, one of these community wells started to produce groundwater with a very high concentration of arsenic and had to be shut down. It is not clear at this point why this happened, but a similar situation was documented for another community well in the area and attributed to a faulty shallow pipe connection (14). A community well was re-installed to the same depth and at the same location in Balia Para and currently produces groundwater that meets the Bangladesh standard for arsenic but not the lower WHO guideline (Table 1).

### Accessing and updating safe-depth thresholds in the field

For access to the BAMWSP database, the unique geo-code of a specific village must first be determined. This is accomplished through a series of short queries sent by SMS that must include at least three sequential letters in the upazila, union, mouza, or village name. The system responds to the query, typically within a few seconds, with the possible names from which the correct administrative units can be selected. The information that is returned for each of these queries can be retained for the next step by replying with the edited text of the previous response. Some flexibility in transliteration of village names from Bangla to Roman letters is allowed because the user can test various combinations of letters. The GPS coordinates can be entered as an alternative to the name-based search. Once the geo-code of a village has been received through SMS, it is sent to the server with a simple command to obtain a summary description of the local test data. The summaries, illustrated in Fig. 4, include the number of wells tested, the proportion of unsafe wells and, when available, the start-depth, together with an estimate of the probability that the estimate is correct. Additional information in the form of a summary of the test data in various depth intervals is also provided. The server automatically adapts the depth intervals of this display to the depth-distribution of the results available for that particular village.

The technology could also allow users to report to the server results for newly-tested wells from the field, together with the location and depth of well. The grey or orange colour of sandy aquifer material encountered during drilling to install a well, which provides a valuable geological context, could be uploaded. Whereas the goal is to provide access to the latest start-depth estimate for anyone using SMS, only certified users, provided with a password, should be allowed to upload new test results or geological information.

## DISCUSSION

### Other constituents of potential health concern

Arsenic is not the only constituent of groundwater that should be considered when installing new wells. The BGS/DPHE (1) survey showed that 35% of the 3,534 well-water samples from across the country that were tested exceeded the WHO guideline value for manganese of 0.5 mg/L at the time. The WHO guideline value for manganese has since then been reduced slightly to 0.4 mg/L (http://www.who.int/water_sanitation_health/dwq/chemicals/manganese.pdf). Exposure to manganese via inhalation is known to be neurotoxic, but little is known about the possible consequences of exposure via drinking-water. A recent study conducted in Araihazar has shown a significant reduction in children's intellectual function at manganese concentrations of >1.0 mg/L compared to children drinking groundwater with <0.2 mg/L (26). The study built on previous work in Araihazar demonstrating that children's intellectual functions were also measurably reduced by drinking groundwater containing arsenic at >10 μg/L (27). Although both arsenic and manganese are naturally released by aquifer-particles under reducing conditions, there is no simple relationship between their concentrations in groundwater in Bangladesh. Many groundwater samples containing little arsenic are enriched in manganese and vice-versa (1, 24). Unfortunately, deeper aquifers that are low in arsenic can still contain significant levels of manganese. Among the 23 community wells that were installed in 10 villages of Araihazar, for instance, seven wells exceeded the new WHO guideline of 0.4 mg/L by up to a factor of nearly 3, counting only once the site where a well had to be re-installed (Table 1). The seven wells elevated in manganese were less than 230-ft (69-m) deep, however. The implication is that, at least in Araihazar, drilling to a depth of >230 ft may increase the chances of reaching groundwater with concentrations of both arsenic and manganese that are sufficiently low.

Several attempts to install community wells in Araihazar were unsuccessful not because groundwater contained too much arsenic or manganese, but because it was too salty due to a pocket of old seawater. Drinking-water typically tastes salty starting at a Na concentration of ∼200 mg/L (i.e. ∼2% seawater), which is the case for six of the 23 community wells installed in Araihazar (Table 1). Even if the salt content of well-water may not be a major health concern, a salty community well will not be used by villagers and will, therefore, not reduce exposure to arsenic. Because of the complex geological history of Bangladesh, it may, therefore, take several attempts in certain villages to reach an aquifer with acceptable levels of arsenic, manganese, and salt.

### Extension to an entire upazila

In this section, we expand the evaluation of potential interventions in the form of community wells to the entire upazila. A closer look into the proportion of unsafe wells in individual villages first reveals that upazila boundaries are arbitrary in terms of arsenic content of well-water. This is because the distribution of arsenic in the sub-surface is determined by the variability of local geology over a range of spatial scales (18, 28). In Araihazar, for instance, the average proportion of unsafe wells of 32% for the entire upazila obscures the fact that villages in the southwestern half of the upazila are much more affected than in the remaining portion (Fig. 1b). Such patterns also suggest that some villages in the upazilas that were not selected for blanket surveying may contain an elevated proportion of unsafe wells.

**Fig. 5.**
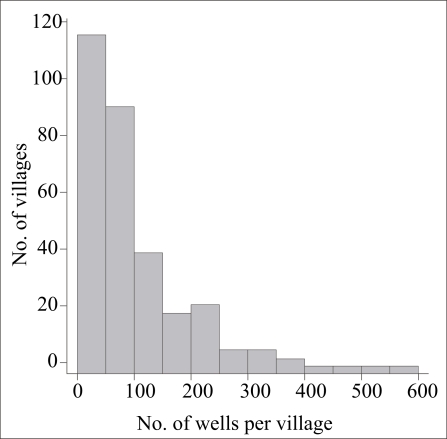
Histogram of the number of wells in 300 villages of Araihazar based on the BAMWSP survey

**Fig. 6.**
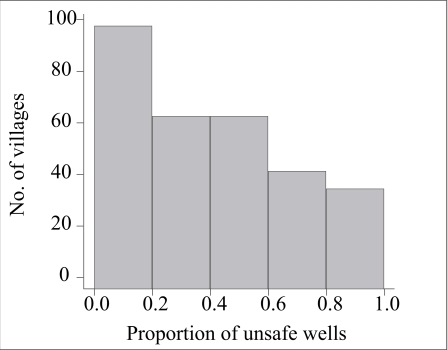
Histograms of proportion of safe wells for ∼300 villages of Araihazar based on BAMWSP data

**Fig. 7.**
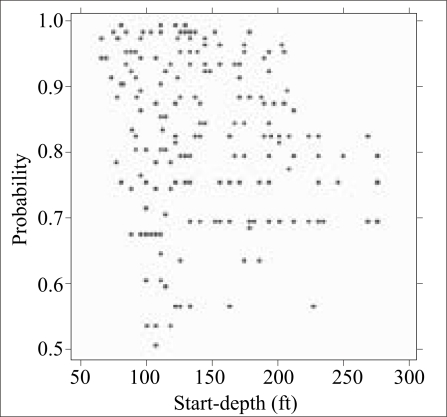
Scatter plot comparing start-depth and proba-bilities that the start-depth is correct for those 261 villages where these parameters could be estimated from BAMWSP data

How was the situation throughout the upazila compared to the 10 villages examined in detail? Most villages of Araihazar are relatively small: 115 of 302 villages (38%) have <50 wells, whereas only 40 villages (13%) have >200 wells (Fig. 5). Less than one-fifth of the wells were unsafe in 99 villages (33%) of Araihazar, while the problem was much more severe in the 35 villages (12%) where over four-fifths of the wells were unsafe (Fig. 6). In 195 villages (65%) of Araihazar, the search algorithm identified a safe-depth threshold of <300 ft and the probability that these safe-depth thresholds are correct ranged from 0.50 to 0.99 (Fig. 7). On the basis of similar conditions encountered in the 10 villages of Araihazar where interventions have already taken place, a local team of drillers using the hand-flapper method should be able to install safe community wells of <300-ft (90-m) depth in most of the 195 villages. The past experience also suggests that the teams should be prepared to drill deeper than the local depth-threshold, however. To maximize the chance of installing a well that meets the WHO guidelines for both arsenic and manganese, drilling should generally continue until aquifer material, i.e. orange-brown in colour, is reached (18, 23). In those cases where such material is not reached, a recently-developed sampling device could be deployed and the groundwater tested before the actual installation of a well (29). The drilling is likely to be disappointing in at least some of the 195 villages identified by the search algorithm, particularly those for which the estimated safe-depth threshold is >200 ft, and/or the probability of the estimate is relatively low (e.g. <0.8). In such cases, a drilling team equipped with a dunkin pump may eventually have to be called in, as was the case in the village of Binair Char.

BAMWSP=Bangladesh Arsenic Mitigation and Water Supply Program

BAMWSP=Bangladesh Arsenic Mitigation and Water Supply Program

BAMWSP=Bangladesh Arsenic Mitigationand Water Supply Program

Two categories of villages were under-represented in the initial set of 10 villages that were studied in detail. These are, on one hand, 65 (22%) of the 302 villages in Araihazar for which the search algorithm could not identify a safe-depth threshold on the basis of the BAMWSP data. For all but three of these villages, the minimum estimated safe depth of <300 ft probably justifies exploratory drilling by a team of local drillers, using the hand-flapper method. The proportion of drilling that turns out to be unsuccessful is likely to be greater than for the previous category of villages for which a safe-depth threshold could be estimated. A third and last category of the classification includes those 42 villages (14%) for which the algorithm has identified a safe-depth of >300 ft. In such cases, a team with a dunkin pump should probably be called in without incurring the expense of drilling a hole with the hand-flapper method. Exceptions could be made for those villages where little or no data are available from past testing for the 200–300-ft range and where a safe aquifer of <300 ft could, therefore, conceivably be identified.

### Prioritizing interventions

**Fig. 8.**
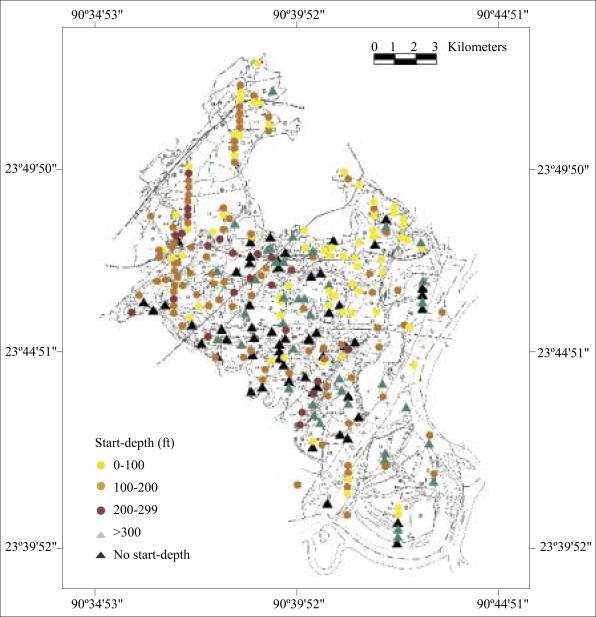
Map showing spatial distribution of start-depths and minimum depths to a safe aquifer throughout Araihazar based on BAMWSP data

**Fig. 9.**
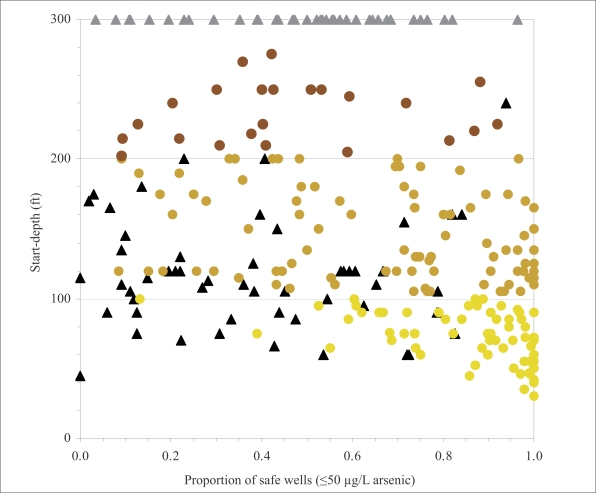
Comparison of start-depths and minimum estimates for start-depths with proportion of unsafe wells in 300 villages of Araihazar. Symbols are same as in Fig. 8.

The goal of interventions in Araihazar is obviously to give access to safe water to the entire population of ∼300,000. However, even if resources were unlimi-ted, it would be important to target the most-affected villages first. In this context, it is worth pointing out that there is no systematic relationship between the start-depths and the proportion of unsafe wells in a village (Figs. 8 and 9). The search algorithm produces a start-depth that is either unidentified or >300 ft (90 m) for many villages with <50% unsafe wells. By the same token, there are many villages with >50% unsafe wells where the algorithm has identified a start-depth of <300 ft. The practical implication is that proportion of unsafe wells and start-depth should be considered when prioritizing interventions.

BAMWSP=Bangladesh Arsenic Mitigation and Water Supply Program

**Fig. 10.**
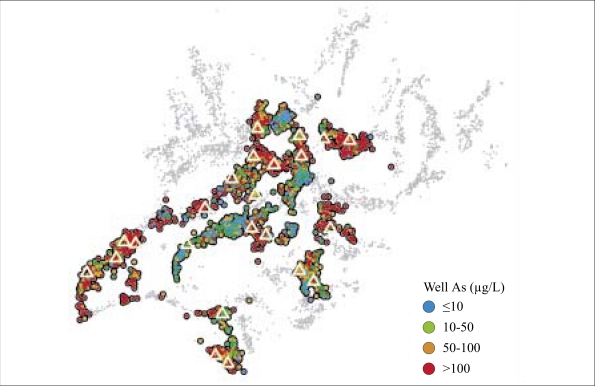
The arsenic content of well-water. The location of community wells is indicated by triangles

The BAMWSP data allow us to estimate the number of wells that are most urgently needed throughout the upazila and how they should be installed. We considered only those villages containing more than 10 wells, which eliminated 10 villages that are likely to be very small or thinly populated out of a total 278 villages for which complete information is available. We would also propose to restrict interventions at first to those villages with a proportion of unsafe wells >50%. Taking into account the geographic extent of the larger villages documented for the portion of Araihazar studied in detail (Fig. 10), we would propose to install one community well for each unit of 100 wells, or fraction thereof, that has been inventoried in a particular village. We also distinguish villages according to the start-depth estimated by the search algorithm. For the 150 villages of Araihazar with an estimated start-depth of <200 ft (60 m), we would propose to limit the intervention at first to the provision of specific advice. The expectation is that, in most cases, households, or communities that are not willing to share existing safe wells will be able to install their own relatively shallow but safe well. No emergency subsidy should probably be provided for these installations. The next category includes those 32 villages with a known start-depth ranging from 200 to 300 ft (60–90 m) and 54 villages whose start-depth is unknown but might be <300 ft (Table 3). Using the criteria listed above to identify the subset of prioritized villages, the installation of 73 community wells should be attempted using the hand-flapper method in this subset of villages. In the prioritized subset of 42 villages where the start-depth is at least 300 ft, instead, a team of drillers equipped with a dunkin pump should be called in to install 27 wells.

**Table 3.** Categories of villages in Araihazar and the number of community wells that would be required to intervene in all villages having at least 10 wells and a proportion of unsafe wells >0.5. The number of community wells required per village was calculated assuming 1 community well per 100 wells or fraction thereof

Village properties	No. of villages (n=278)	No. of community wells (n=132)	Action
Start-depth <200 ft	150	32	Provide information
Start-depth 200–300 ft	32	39	Install using hand-flapper
Start-depth >300 ft	39	20	Install using dunkin pump
No start-depth but <300 ft	54	34	Explore using hand-flapper
No start-depth and >300 ft	3	7	Explore using dunkin pump

### Installing community wells throughout the country

There is no technical difficulty involved in storing, retrieving, and updating the BAMWSP data for additional upazilas now that the mobile-phone technology has been developed for one upazila. Doing so for the entire BAMWSP dataset could, therefore, immediately help the Bangladesh Department of Public Health Engineering (DPHE) optimize its ongoing programme of installations of wells throughout the country. How many wells would have to be installed to reach coverage comparable to that estimated above for Araihazar? On the order of 17,000 wells, considering that 50,000 villages distributed over 250 upazilas have been surveyed under the BAMWSP. The assumption is that the distribution of arsenic over the entire area is broadly similar to that of Araihazar. The process would take over a decade, however, at the current rate of installation of ∼1,000 wells per year under the current DPHE structure. The possible implication is that the DPHE should focus its efforts on installing community wells by contracting teams of drillers using a dunkin pump, while leaving installations by local teams of drillers using the hand-flapper method to a different type of organization. The decentralized structure of a large NGO, such as BRAC, might be more suitable for supervising installations of wells relying on local teams of drillers in those villages where this approach is likely to be successful. If the mobile-phone technology could be systematically used by the DPHE and NGO workers involved in installations of wells to upload sand colour, well-depth, and field-kit results, the national dataset would become increasingly valuable over time and reduce the risk of installing unsafe wells.

This analysis of existing data from Araihazar upazila illustrates how the BAMWSP data interpreted at the village scale can guide the installation of safe community wells. SMS-based mobile-phone technology can provide an inexpensive and convenient means of accessing and, importantly, updating the BAMWSP dataset from any location. Start-depths calculated on the basis of up-to-date information could help choose the drilling technology and the type of organization that is most suited to the installation of safe community wells throughout the country.

## ACKNOWLEDGEMENTS

The mobile-phone technology was developed with support from the Earth Clinic of the Earth Institute at Columbia University. Columbia University and the University of Dhaka's research and mitigation work in Araihazar since 2000 has been supported by NIEHS Superfund Basic Research Program grant no. NIEHS 1 P42 ES10349, Fogarty International Center grant no. NIEHS 5-D43 TW05724-01, NSF grant no. EAR 03-45688, NSF grant no. EAR 04-33886, and the Earth Institute at Columbia University. The authors thank their colleagues at the Mailman School of Public Health, Lamont-Doherty Earth Observatory, and the Earth Institute, particularly Joseph H. Graziano, Habibul Ahsan, Yan Zheng, Martin Stute, and Alex Pfaff, for their contributions to mitigation in Araihazar over the years. It was Jeffrey Sachs, Director of the Earth Institute at Columbia University, who challenged the authors to consider mitigation beyond their immediate study area. This would have been foolhardy without the benefit of Mushtaque Chowdhury (BRAC)'s active participation in group discussions that led to the approach to mitigation presented in this paper. Finally, the authors would like to thank the team of drillers, led by Abu Taleb and Shaidullah, for their tireless work.
